# A Text Book of Operative Dentistry

**Published:** 1889-05

**Authors:** 


A TEXT BOOK OF OPERATIVE DENTISTRY.—By Thomas Fillebrown, M. D., D. D. S., Professor of Operative Dentistry in Harvard University Dental School, Etc., Etc.

Written by invitation of the National Association of Dental Faculties. Two hundred and seventy-three pages, and three hundred and thirty illustrations. Philadelphia: P. Blakiston, Son & Co., Publishers, 1012 Walnnt St. Price $2.50.

Dr. Fillebrown can congratulate himself on the success of his effort to produce a text-book on Operative Dentistry that is confined more especially to the descriptions of the manual operations required for the preservation of the natural teeth. The author announces in his preface that his work is not intended as a substitute for larger works, but as an epitome of the practical application of the principles discussed at length in more extensive volumes and to these he refers the student for exhaustive discussion. The more advanced nomenclature has been adopted as far as professional sentiment would sustain the author. The work contains many valuable suggestions presented in a concise manner, and is a valuable addition to dental literature.

